# Raman spectroscopy as a process analytical technology for pharmaceutical manufacturing and bioprocessing

**DOI:** 10.1007/s00216-016-9824-1

**Published:** 2016-08-04

**Authors:** Karen A. Esmonde-White, Maryann Cuellar, Carsten Uerpmann, Bruno Lenain, Ian R. Lewis

**Affiliations:** 1 Kaiser Optical System, Inc, 371 Parkland Plaza, Ann Arbor, MI 48103 USA; 2 University of Michigan Medical School, Ann Arbor, MI 48109-5624 USA; 3 Kaiser Optical Systems SARL, 5 Allée Moulin Berger, 69130 Ecully, France

**Keywords:** Raman spectroscopy, Pharmaceutical, Process analytical technology, Bioprocessing, Cell culture, Recombinant protein

## Abstract

Adoption of Quality by Design (QbD) principles, regulatory support of QbD, process analytical technology (PAT), and continuous manufacturing are major factors effecting new approaches to pharmaceutical manufacturing and bioprocessing. In this review, we highlight new technology developments, data analysis models, and applications of Raman spectroscopy, which have expanded the scope of Raman spectroscopy as a process analytical technology. Emerging technologies such as transmission and enhanced reflection Raman, and new approaches to using available technologies, expand the scope of Raman spectroscopy in pharmaceutical manufacturing, and now Raman spectroscopy is successfully integrated into real-time release testing, continuous manufacturing, and statistical process control. Since the last major review of Raman as a pharmaceutical PAT in 2010, many new Raman applications in bioprocessing have emerged. Exciting reports of in situ Raman spectroscopy in bioprocesses complement a growing scientific field of biological and biomedical Raman spectroscopy. Raman spectroscopy has made a positive impact as a process analytical and control tool for pharmaceutical manufacturing and bioprocessing, with demonstrated scientific and financial benefits throughout a product’s lifecycle.

## Introduction

Raman spectroscopy is an optical spectroscopy technique that provides a “molecular fingerprint” of a sample. As an optical method, Raman enables nondestructive analysis of chemical composition and molecular structure. Applications of Raman spectroscopy in polymer, pharmaceutical, bioprocessing, and biomedical analysis have surged in the past three decades as laser sampling and detector technology has improved. Because of these technological advances, Raman spectroscopy is a practical analysis technique inside and outside the laboratory. Raman spectroscopy is an established process analytical technology (PAT) tool. Since the 1980s, Raman spectroscopy has been used to study active pharmaceutical ingredients (API). Raman spectroscopy as a tool for API analysis has been described for many applications, including polymorph identification, quantitative analysis, in situ crystallization monitoring, real-time release testing, pharmaceutical unit operations, and process-induced transformations [1–5]. In addition to identifying isolated polymorphic forms, mixtures of forms can be analyzed and quantified [6, 7]. The diverse structures that have been measured by Raman, from the discovery laboratory to the manufacturing environment, show that Raman can reliably provide quantitative data. In-line Raman spectroscopy can control critical process parameters, enables real-time process corrections, and ensures consistent production of the correct API form. We highlight new applications in API synthesis and crystallization, real-time release testing, flow or continuous manufacturing, and new developments in Raman spectroscopy for understanding and controlling bioprocesses.

## Regulatory perspectives and guidance

A philosophical shift in pharmaceutical manufacturing quality, which is strongly encouraged by regulatory agencies, has created opportunities to integrate real-time process analytics into manufacturing processes. In 2002, the U.S. Food and Drug Administration (FDA) launched an initiative to encourage innovation in manufacturing technology and quality system approaches. The FDA 2004 PAT framework strongly emphasized a shift from tested-in quality after the drug product was produced to building in quality *throughout* production with “continuous real time quality assurance” [8]. The European Medicines Agency (EMA) established a PAT team in 2003, which released guidance documents on process PAT, quality by design (QbD), and real-time release testing. International Conference on Harmonization (ICH) Q8, Q9, Q10, and Q11 documents reinforced FDA and EMA guidance, which has been implemented in the USA, European Union, and Japan since 2009. Importantly, the FDA and ICH documents provided a strategic guidance, rather than prescriptive guidance, on developing an approach to understand and manage risks that might affect critical quality attributes. PAT has an important role in this new framework to understand and manage risk throughout a pharmaceutical product’s lifecycle. Recently, these principles were extended to bioprocessing. As a PAT in pharmaceutical manufacturing and bioprocessing, Raman spectroscopy has demonstrated value from scientific understanding to process control.

## Instrumentation and data analysis techniques

Over the past 25 years, Raman spectroscopy instrumentation has evolved from home-built academic laboratory instruments to robust commercially available solutions-based systems. The advent of stable laser sources, high-speed optical fibers, volume holographic gratings, and low-noise charge coupled device detectors enabled robust commercial Raman spectroscopy instrumentation. Newer commercial instruments are straightforward to use because they do not require constant realignment or sophisticated knowledge of optics, are equipped with instrument control software, and are integrated with Raman spectral libraries. Thus, Raman spectroscopy is accessible to scientists and environments beyond the academic research environment.

Modern instrumentation has been reviewed in detail elsewhere [9–11]. Briefly, there are three basic components of a Raman spectrograph, including a laser, sampling optics, and detector. We will focus on laser wavelength and fiber optic sampling probes in terms of their impact on Raman spectroscopy as a PAT. Inelastic scattering is a weak phenomenon, occurring only in a small amount of photons. Modern Raman instrumentation optimizes the amount of inelastically scattered photons and their detection. Modern Raman instruments use a laser as the illumination source because it is a high-intensity monochromatic source of light. While the laser wavelength can vary from the UV to the near-infrared (λ = 200–1064 nm), most pharmaceutical or bioprocessing applications use near-infrared wavelengths (λ = 785 or 830 nm), primarily to minimize fluorescence interferences. For example, in Chinese hamster ovary (CHO) cell culture bioprocesses, autofluorescence from intracellular NADH and flavins occur in visible wavelengths and the strong autofluorescence necessitates Raman measurements in the near-infrared wavelengths (λ = 785 nm or 830 nm) even though the scattering efficiency is lower in the near-infrared compared with UV and visible wavelengths [12, 13].

Although near-infrared wavelengths are used primarily to minimize interference from fluorescence, there are other benefits that are not as widely recognized. All light-cell (or particle) interactions are important considerations in choosing a wavelength and sampling optics for pharmaceutical manufacturing and bioprocessing applications. Raman scattering occurs in the presence of fluorescence, optical scattering, and optical absorbance. Optical scattering and absorption effects are becoming increasingly important to understand, especially as Raman spectroscopy moves toward in situ measurements in turbid media. Most cell and tissue spectroscopy is performed in the “therapeutic” near-infrared window (650–1350 nm) because optical scattering is dominant and water absorption is at a minimum [14, 15]. The same principle applies to pharmaceutical or bioprocess measurements, where it is desirable to minimize optical absorption. Optical absorption can arise from water, media chromophores, or a pigmented cell. Optical scattering arises from refractive index mismatches. Particles, bubbles, or droplets with sizes approaching the excitation wavelength exhibit Lorenz-Mie scattering, which causes aqueous systems to become turbid. Photons can be scattered multiple times, resulting in photons being diffusely distributed in a turbid media. API or excipient particles and cellular organelles, such as mitochondria and nuclei, also strongly scatter light [16, 17]. Understanding photon transport in turbid media is an important consideration for quantitative Raman spectroscopy applications in content uniformity, real-time release testing, and in situ bioprocess control.

Sampling optics may be a microscope for high spatial resolution measurements, wide area or transmission for bulk measurements, or an immersion optical fiber probe for in-process measurements. Fiber optic probes can be interchanged on an instrument, thus extending the capabilities of a Raman instrument. For example, a single Raman instrument can be equipped with a microscope, a probe optimized for solids identification, and a probe optimized for in situ reaction monitoring. This sampling versatility is an attractive feature as a PAT, especially in-process development and technology transfer. As an optical spectroscopy technique, Raman spectroscopy can be performed outside of the laboratory using fiber optic instrumentation. An important question when selecting a fiber-optic probe for measurements in turbid media or solids is the desired sampling volume where optical scattering is significant.

**Fig. 1:**
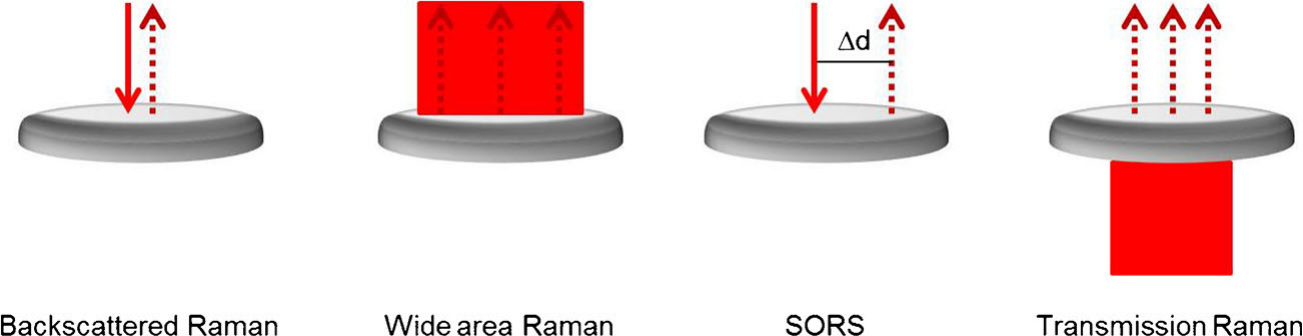
Schematic comparing variants of sample excitation (solid line) and signal collection [dashed line(s)] used in Raman spectroscopy in measuring turbid media. (**a**) Backscattered Raman is a commonly used geometry that uses a single site of excitation with collection of signal close (<1 mm) to the excitation. As applied to Raman microscopy, this approach is called epi-illumination as a single microscope objective is used to excite the sample and collect Raman signal. The sampling volume in backscattered Raman is generally small, both in the lateral and axial dimensions. Thus, backscattered Raman is a good approach for measuring a surface. (**b**) Overlapping a defocused or wide laser beam with multiple collection fibers in a backscattering-like geometry is called wide area Raman (also known as large volumetric or global illumination Raman). (**c**) Separating collection fibers from the sample excitation by a small distance (Δd = 1–6 mm) enables collection of diffusely scattered Raman photons, known as a spatially-offset Raman spectroscopy (SORS). (**d**) Transmission Raman collects Raman photons diffusely scattered through a sample. Wide area, spatially-offset Raman spectroscopy (SORS) and transmission Raman provides representative sampling in turbid media and enables collection of Raman signal from buried layers in a layered sample

Much research has been devoted in developing Raman spectroscopy for pharmaceutical solids analysis, taking into consideration process compatibility, validation, and ease of use. Figure 1 shows the variants of Raman spectroscopy that utilize fiber optic probes. Traditional approaches employed fiber configurations with minimal separation between the excitation and collection fibers (Fig. 1a). This technique is called backscattered Raman, and the signal collected is mainly from superficial layers with minimal or no signal recovery from subsurface layers. Raman microscopy uses an epi-illumination configuration, with the microscope objective both delivering focused laser light to the sample and collecting Raman-scattered photons. Wide area Raman, also called large volumetric or global illumination Raman, (Fig. 1b) utilizes a defocused laser beam to illuminate a large area within the sample. The collection and illumination areas are completely overlapping, and this approach has been shown to be an effective means of sampling superficial and deep layers, and improves upon non-representative (or sub-sampling) issues encountered using backscattered Raman [18, 19]. Extension of diffuse reflectance principles and fiber-probe designs to Raman spectroscopy is called spatially-offset Raman spectroscopy (SORS). SORS employs a larger (1–3 mm) separation between the illumination and collection fibers (Fig. 1c). The fiber separation enables collection of subsurface signal, even through millimeters or centimeters of turbid media, because Raman-scattered photons from subsurfaces are diffusely scattered afield from the illumination fiber [20, 21]. The fiber separation can be tailored to optimize recovery of subsurface signal and reduce fluorescence interferences from the superficial layer. Excitation of a sample using a defocused laser, and collection of Raman signal through a sample is the basis for transmission Raman (Fig. 1d) [22–24]. Transmission Raman also provides representative sampling and suppresses fluorescence from superficial layers, rapidly providing a bulk Raman measurement of a pharmaceutical formulation [25, 26]. Backscattered, wide area, and SORS can be used in-line, on-line, at-line, or off-line, whereas transmission Raman is commonly used as an off-line PAT.

Two other configuration variants deserve mention: Raman tomography and enhanced reflection Raman. Over a large anatomic site, such as a leg or wrist, backscattered, diffusely scattered, and transmitted Raman signal can be simultaneously measured at multiple locations to generate a 3D image. This approach, known as Raman tomography, noninvasively provides dimensional, anatomic location and chemical information. Raman tomography was developed in 2008 as an academic research tool for transcutaneous measurement of bone, with potential application in pharmaceutical tablet analysis [27–29]. Enhanced reflection is the addition of a reflecting mirror, integrating sphere, or photon diode, to backscattered, SORS, or transmission configurations to improve signal recovery and selectivity in sampling thin layers. This approach is effective because elastically scattered photons can be reflected back into the sample and possibly undergo inelastic scattering [30]. Enhanced reflection Raman coupled into a wide area configuration improves layer selectivity and provides a bulk measurement similar to transmission Raman [31, 32] and improves selectivity in thin film measurements in transmission Raman [33]. The technique is easy to implement and does not require engineering modifications to equipment. These features give enhanced reflection Raman an advantage over transmission Raman, which is difficult to implement as an on-line PAT, for the purposes of on-line process control by Raman spectroscopy.

There are two main approaches to analyzing Raman data: univariate and multivariate. A univariate approach uses Raman band features of area, intensity, or center of gravity to understand the sample chemistry. Most univariate Raman data are reported as band ratios, where band intensities or areas are ratioed. Band ratios have been correlated to a material’s mechanical properties, chemical composition, or a pharmaceutical solid’s crystal form. Although a univariate data analysis is straightforward to employ, it requires that the components of interest have distinguishing and unique Raman bands. Important bands overlap in biological tissue, pharmaceutical formulations, or bioprocesses, and use of multivariate data analysis techniques is required. Multivariate data analysis, or chemometrics, is widely used in biomedical Raman spectroscopy [34], pharmaceutical imaging [35], and process analytical technology [36]. Partial-least squares (PLS) and principal components analysis (PCA) are the commonly used models in pharmaceutical and bioprocessing [37]. Model transferability, validation, and robustness are important considerations, regardless of model type. Criteria to assess model suitability may include standard error of calibration (SEC), standard number of factors used, and coefficient of determination (R^2^). However, many variations of model suitability criteria have been reported, depending on the manufacturing process.

## Considerations in process Raman spectroscopy

Within the process environment, the sampling flexibility of Raman spectroscopy means that Raman can be employed as an off-line, at-line, on-line, or in-line (or in situ) PAT. There are additional logistical considerations when translating Raman spectroscopy into a process environment. Two reviews provide a comprehensive list [38, 39]. Data integrity and compatibility with control systems or quality risk management systems require additional software engineering. Process conditions, chemical compatibility of immersion probes, environmental conditions, and operator safety need to be reviewed thoroughly before implementing any PAT. A process-specific discussion of these considerations is provided by Hart et al., for a heterogeneous etherification reaction [40]. The manufacturing process may require additional supplier qualification, especially if an excipient’s material attribute is found to be a critical process parameter [41].

## Pharmaceutical manufacturing

Recent years have brought a sea of change in pharmaceutical manufacturing. Regulatory support, combined with recognized scientific and financial benefits, are major factors in the widespread adoption of QbD and PAT [42–44]. The philosophical shift to QbD has encouraged new risk-based approaches in real-time release testing, continuous manufacturing, and statistical process control. Real-time, in-process analytics have an important role in ensuring quality product and enabling in-process corrections. Focused-beam reflectance, infrared, near-infrared, and Raman spectroscopies are attractive as in-process analytics because they rapidly and nondestructively provide chemical and physical properties information. The sharp Raman spectral features and compatibility with aqueous environments are attractive features for in-process measurements. For many years, these features have been harnessed to understand pharmaceutical small molecule crystallization and processing.

## API reaction analysis

**Fig. 2:**
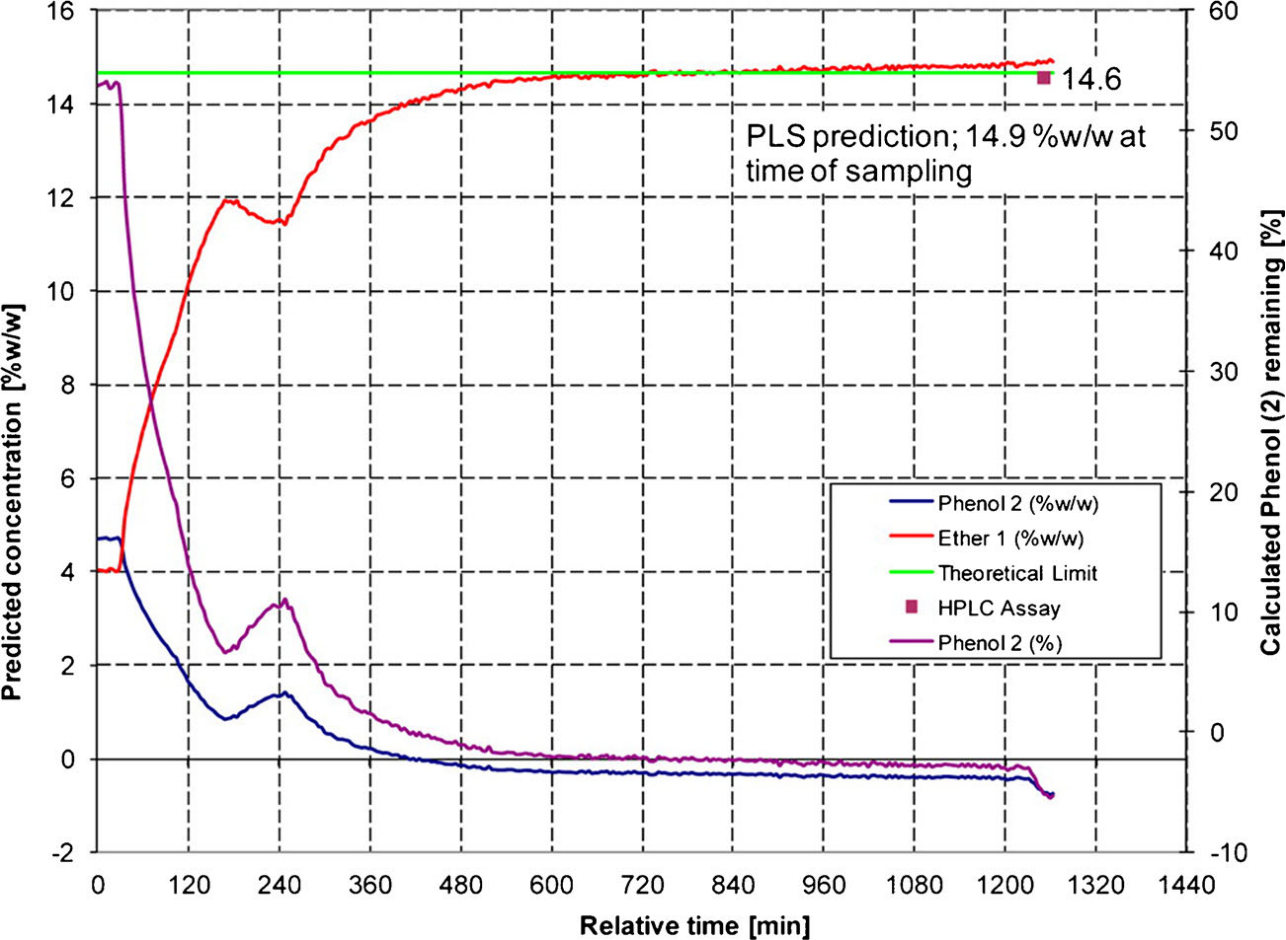
A time course of predicted Raman concentrations of phenol and ether for a second pilot plant batch, with off-line HPLC and theoretical limit of ether. In situ Raman spectroscopy was used to control a heterogeneous etherification reaction, with Raman measurements of ether %w/w used to predict end of reaction. Reagent was added from 0 to ~180 min followed by a line wash, which accounts for the profile disturbance at ~ 240 min. In the process description and batch sheet, the reaction would have been completed at ~ 1250 min and at that time a sample would be collected for offline HPLC analysis (square in figure). In situ Raman data showed reaction completion nearly 600 min before stipulated time. The data suggest that batch cycle time could be reduced by several hours when moving up to the commercial manufacturing scale, improving process efficiency. Reprinted with permission from Hart, Richard J., Nicholas I. Pedge, Alan R. Steven, and Kevin Sutcliffe. “In Situ Monitoring of a Heterogeneous Etherification Reaction Using Quantitative Raman Spectroscopy.” Organic Process Research & Development 19, no. 1 (January 16, 2015): 196–202. doi:10.1021/op500027w. Copyright 2016 American Chemical Society

Raman spectroscopy has an important role in understanding and controlling the manufacture of an active pharmaceutical ingredient (API). In-process Raman for reaction monitoring and analysis during unit operations have been reviewed [6, 45]. Since those reviews, there has been interest in extending the capabilities of Raman in monitoring highly exothermic, heterogeneous, or continuous flow reactions. An example shows how Raman, NIR, FBRM, UV/Vis, and particle vision were integrated into a single intelligent decision support system [46]. Another illustrative example shows Raman-based control of a model exothermic oximation reaction, which successfully controlled accumulation of an unstable intermediate [47]. Hart et al. published a comprehensive paper on the many considerations in using a PAT for API synthesis [40]. In that study, a heterogeneous etherification reaction involving chloropyrazine and phenol in the synthesis of Compound X was monitored by Raman spectroscopy. Raman predictions were used to determine the reaction endpoint. The method and data model were developed at the laboratory scale (250 mL). During method development, solvent charges, different batches of input materials (to model variable fluorescence background), and different baseline correction routines were tested. The end of reaction was based on %w/w ether. Two pilot plant scale (1500 L) reactions were reported. In the first pilot plant batch, Raman predictions were shown to be equivalent to HPLC. Only Raman was used as the analytical control in the second pilot plant batch. As shown in Fig. 2, Raman predictions of %w/w ether showed that the reaction was completed 600 min before the batch sheet and process description stipulations, indicating more efficient mixing at pilot scales.

In addition to providing scientific results and discussion, the authors discussed logistical factors that informed on their decisions in adopting an on-line PAT strategy. Based on our experience, the myriad considerations described in the paper are representative of the industry. The authors discussed how economic, scientific, and risk management factors were integrated into a control strategy using on-line Raman spectroscopy. The main economic driver was to reduce batch cycle time, resulting in a cost of goods savings of the API. Scientifically, on-line Raman addressed concerns that the off-line HPLC sample was not representative of the heterogeneous reaction mixture. From a risk management perspective, on-line Raman can be used to troubleshoot if the reaction endpoint was not achieved, and allowed reaction profiles to be monitored by chemists 200 miles away.

Pharmaceutical excipient chemical and physical properties are typically a critical process parameter because they affect manufacturability, bioavailability, and risk of process-induced API transformations. Raman spectroscopy measures excipient material attributes nondestructively and rapidly, with handheld systems typically used for this application. A comprehensive database of commonly used pharmaceutical excipients contains both the Raman spectrum and band assignments [48]. The excipient spectrum can be affected by different crystal forms, amorphous content, or process variations. In-house preparation of excipients or biopharmaceutical formulations may require its own risk-based manufacturing approach [49]. For example, the crystallization and drying steps in the preparation of sodium carbonate, an excipient in effervescent tablets, were shown to have a high impact on the tablet’s performance. Sodium carbonate production, by batch or continuous processing, was monitored by Raman spectroscopy, laser diffraction, and X-ray powder diffraction as part of ensuring a quality excipient [41].

## Real-time release testing

The PAT and QbD initiatives marked a shift away from end-process release testing to real-time release testing (RTRT), defined in ICH Q8(R2) as “the ability to evaluate and ensure the quality of in-process and/or final product-based on process data” and typically uses a combination of controlling process parameters and monitoring product attributes. A RTRT strategy may include at-line chemical and physical property measurements of raw materials, spectroscopic monitoring of concentration and uniformity during blending, particle size distribution measurement after granulation, imaging or spectroscopy of coating, and a multivariate dissolution model [50]. Within the context of RTRT, Raman spectroscopy has made important contributions as a PAT in pharmaceutical unit operation of blending, granulation, tableting, and coating. We focus on new research in in-line or off-line Raman measurements of content uniformity and tablet coating.

Raman PAT tools are robust to particle size and provide representative sampling. Tablets and capsules are the most common pharmaceutical formulation, which is reflected in the number of Raman reports on tablets or capsules. As an off-line PAT, transmission Raman and micro-scale wide area Raman rapidly and nondestructively provides API measurements in tablets. Pharmaceutical applications of transmission Raman consistently report rapid API quantification with suitable prediction model error, and significant reduction of fluorescence or tablet coating signal [24, 25]. As a first screen of content uniformity in pharmaceutical tablets or capsules, transmission Raman can measure API distribution, polymorph impurities, to 0.1 %, and insoluble excipients that cannot be measured using HPLC. It is possible to obtain even lower API quantification levels using wide field Raman. Using a micro-scale wide field Raman mapping instrument, Li et al. recently demonstrated trace API quantification, with limits of detection below 0.1 %. With a prediction accuracy of 2.4 %, Raman provided quantification to 0.03 % similar to 0.041 % by HPLC [51]. Application of transmission Raman to quantify API in a bilayer tablet was reported by Zhang and McGeorge [33]. A modified Kubelka-Munk model of optical scattering and photon transport showed that the measured API Raman signal was a function of API concentration, and thickness of API layer and thickness of excipient layer. A Design of Experiment approach to experimentally verifying model predictions showed that each layer had different optical scattering properties so that tablet orientation had an effect on the photon attenuation coefficient and recovery on sublayer Raman signal. A transreflectance configuration improved upon layer selectivity and enhanced signal 6-20 fold.

Low levels of polymorph impurities were examined in the laboratory on a model tablet using NIR, backscattered, and transmission Raman spectroscopies [52]. Both backscattered (200 μm spot size) and transmission Raman (8 mm spot size) were better than near-infrared at identifying low levels (0.6–0.7 %) of a polymorph impurity in a simulated tablet model. Transmission Raman measured more volume of the table more rapidly and with less model prediction error (RMSEC = 0.29, R^2^ = 0.998) than backscattered Raman (RMSEC = 1.11 R^2^ = 0.965). Backscattered Raman measurements were collected at 16 sites on the tablet and the data were averaged to avoid sub-sampling but this approach does not address sub-sampling within the tablet volume since backscattered Raman measures surfaces with minimal contributions from deeper layers. Essentially, the authors did not control for differences in the sampling volume between backscattered Raman and transmission Raman. A study of pharmaceutical capsules using confocal Raman microscopy and transmission Raman also directly compares instrument performance without controlling for differences in sampling volume [25]. A more direct comparison would be of the two variants, which employ a defocused laser: wide area and transmission. Photon transport in wide area and transmission Raman were modeled using Monte Carlo and experimental validation of the model [22, 31]. Both studies reported that transmission Raman enabled quantification of small quantities in deep layers, and backscattered Raman was highly surface-specific. Incorporation of a reflecting surface into the wide area setup, such as a reflective belt or station, increased the ability of wide area Raman to probe in deep layers. Although transmission and enhanced reflection Raman provided similar bulk measurements, enhanced reflection can be used as an in-line PAT while transmission Raman is an off-line PAT.

Content uniformity is challenging to measure in non-oral dosing formulations where heterogeneity in API distribution may affect bioavailability. A recent example highlights how the spatial resolution and chemical specificity of Raman spectroscopy can be used to understand multiple content uniformity parameters such as API concentration and spatial heterogeneity. Wide area Raman spectroscopy of dapivirine in a polymeric controlled release device predicted API values, assessed initial process capability, and demonstrated that heterogeneous API distribution in the device would not affect product specifications [53]. Another example of measuring spatial heterogeneity in API distribution was reported by Baronsky-Probst et al. where off-line Raman imaging was used to measure API distribution in a hot melt extrusion [54].

Raman has demonstrated value as an in-line PAT for batch or continuous tablet coating processes, providing both process control and real-time coating release [55, 56] and capable of meeting ICH Q2 guidelines for an active coating process [4].

## Flow or continuous manufacturing

Intense reaction conditions, non-traditional chemistries, and miniaturized reactors are hallmarks of continuous manufacturing. One of the earliest applications of continuous reaction monitoring was reported in 1997, where on-line Raman spectroscopy was used to measure the continuous reaction between phosphorus and chlorine to produce phosphorus trichloride [57]. Owing to the “boiling, toxic, pyrophoric, and corrosive reaction mixture,” an on-line reaction analysis tool was needed. On-line dispersive Raman was selected to replace on-line FT-Raman. Raman spectroscopy was able to directly measure all components of interest throughout the reaction, was sensitive to better than 1 % for reactants and products, and provided fast feedback. Continuous manufacturing is commonly used in chemical and petrochemical industries, with pharmaceutical applications only recently realized. Extension of continuous manufacturing principles to the pharmaceutical industry is now applied from primary API processing to “powder to tablet” secondary processing [58, 59].

On-line PAT in continuous manufacturing reactors eliminates the need for slow off-line analyses, and improves the ability to make timely process control decisions. Feasibility studies show that Raman spectroscopy can be used to monitor continuous heterogeneous catalysis reactions [60] and catalytic oxidation reactions [61] in a microfluidic reactor (or microreactor). Roberto et al. reported on-line Raman monitoring of an esterification of benzoic acid in the NeSSI sampling system [62]. Incorporation of a PLS model enabled predictions of chemical conversion of benzoic acid to methyl benzoate. Mid-infrared and Raman combined with PLS and multivariate statistical process control (MSPC) to provide real-time quality assurance for flow synthesis of an oligonucleotide [63]. Raman, X-ray powder diffraction, and laser diffraction were used to monitor continuous or batch production of sodium carbonate, an excipient in effervescent formulations [41]. These examples demonstrate that Raman is an important on-line PAT in continuous synthesis. In secondary manufacturing, Fonteyne et al. reported successful prediction of residual moisture content using on-line Raman and near-infrared and prediction of granule flowability using photometric imaging during a continuous granulation production [64].

## Bioprocessing

Raman spectroscopy of biological molecules has a rich history. The well-known benefits of Raman spectroscopy, including sharp spectral features that correlate to a sample’s chemical or molecular structure properties, nondestructive nature, and compatibility with aqueous systems, are attractive features for tissue and cell culture studies. Mineralized biotissues, oligomers, polypeptides, and proteins were among the first biological species examined by Raman spectroscopy [65, 66]. Raman studies into collagen and globular proteins provided insight into the protein’s secondary structure and complemented circular dichroism and X-ray diffraction studies. Raman studies of fatty acids, amino acids, polysaccharides, metabolites, carotenoids, nucleic acids, and glycoproteins soon followed. There are excellent reviews providing Raman spectra of biomolecules, complete with tables of band assignments [66, 67]. Biological and biomedical Raman applications emerging in the 1990s had an emphasis on histopathology, disease diagnosis, biological tissue examination, and in vivo glucose monitoring [68–70]. Concurrent to biomedical application development, there was an effort to use Raman to understand metabolism during fermentation or cell culture bioprocesses.

At the time on-line Raman of bioprocesses was first reported in the late 1980s, there was strong literature in Raman spectroscopy of amino acids, metabolites, alcohols, and polysaccharides. Raman spectroscopy was understood to be a viable choice for in situ bioprocess monitoring but there were a limited number of published reports, and literature reviews in 2004 and 2010 reflected that understanding [10, 71]. Since those reviews, new technology has enabled industrial applications, and, as a result, many papers have emerged since 2010 that demonstrate successful application of Raman spectroscopy to monitor and control bioprocesses. In situ, simultaneous measurement of nutrients, metabolites or by-products, cell density (or biomass), and method transferability are features that have made Raman spectroscopy an important PAT in bioprocessing.

The first bioprocess studied by Raman spectroscopy was fermentation, and there is ongoing research to further optimize in situ quantification. Shope et al. reported in 1987 on-line Raman measurement of fermentation components such as methanol, ethanol, and acetone [72]. Other reports include off-line Raman measurements of glucose, glutamine, lactic acid, and ammonia drawn from a cell culture bioreactor [73], or in-line Raman measurements of ethanol, glucose, and fructose of *Saccharomyces cerevisiae* during alcoholic fermentation [74]. The first FT-Raman study in 2001 reported off-line measurement of glucose, ethanol, and cell density of *S. cerevisiae* during ethanol fermentation, with the goal of process control [75]. Another early in-line Raman study reported direct measurement of carotenoid production by *Phaffia rhodozyma*, and the Raman data were used to determine an optimal feeding strategy [76]. In these early studies, academic researchers used laboratory equipment or custom-built fiber optic probes to perform their measurements.

**Fig. 3:**
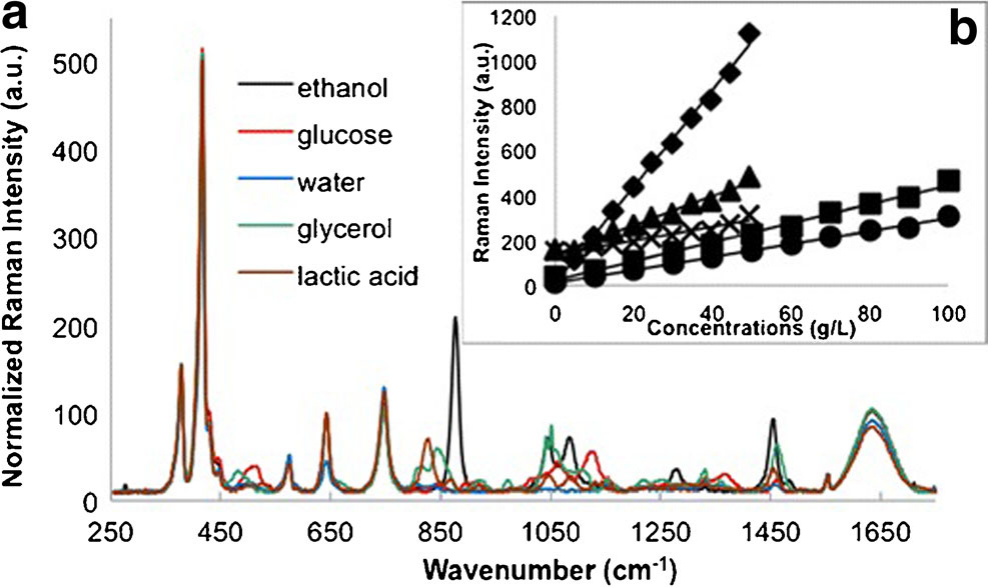
(**a**) Raman spectra after fluorescence corrections of reference components: ethanol, glucose, water, glycerol, and lactic acid, and (**b**) standard plot of baseline-corrected spectra for a simulated fermentation of 100 g/L glucose up to 50 g/L. Ethanol measured at 877 cm^−1^ (diamond), y = 21.406x + 12.553, R^2^ = 0.9959; 1046 cm^−1^ (multiplication sign), y = 3.0716x + 138.07, R^2^ = 0.9569 at 1455 cm − 1 (triangle), y = 6.4871x + 138.52, R^2^ = 0.984. Glucose measured at 514 cm^−1^ (circle), y = 2.8793x + 11.362, R^2^ = 0.9973, and 1,123 cm^−1^ (square), y = 4.2116x + 24.177, R^2^ = 0.994. Reprinted with permission from Iversen, Jens A., Rolf W. Berg, and Birgitte K. Ahring. “Quantitative Monitoring of Yeast Fermentation Using Raman Spectroscopy.” Analytical and Bioanalytical Chemistry 406, no. 20 (2014): 4911–4019. doi:10.1007/s00216-014-7897-2. Copyright 2014 Springer.

Recent research in Raman spectroscopy of fermentation bioprocesses have developed novel fiber optic probes and refined data analysis models, with the goal of in situ quantification of important parameters such as glucose, ethanol, and cell concentration. In situ Raman quantification of glucose, ethanol, and yeast concentrations during a *S. cerevisiae* fermentation was reported by Picard et al. in 2007 and Iversen et al. in 2014 [77, 78]. In the study by Iversen et al., a specially-designed probe delivered 785 nm light and collected Raman signal. The probe was directly inserted into the 1 L bioreactor, enabling in situ Raman measurements. Raman predictions were compared with HPLC or UV/Vis reference measurements. The authors performed reference measurements of pure component ethanol/glucose mixtures (1:1 v/v 70 g/L ethanol:20 g/L glucose) while varying yeast cell concentrations in order to understand the effect of optical density on Raman signal attenuation. Raman spectra of pure components and model results are shown in Fig. 3. From the optical scattering experiments, the authors found a non-uniform baseline shift. The oscillating pattern in the Raman spectra was consistent with yeast cell concentration. Proposed sources of the oscillation pattern were changes in angular scattering intensity, partial Mie scattering, or resonance fluorescence. Further inspection on the effect of increasing optical density showed significant variance in the response of analyte’s Raman signal, but little effect on fluorescence. For example, the ethanol band at 877 cm^–1^ was attenuated more quickly than the glucose band at 1123 cm^–1^ or the water band at 1627 cm^–1^ with yeast cell concentration. Because a general extinction relationship could not be calculated, a relationship was developed for each component: glucose, ethanol, and cell biomass. Correction of Raman signal attenuation resulted in marked improvement to the root mean square error of prediction over non-corrected data for glucose and ethanol. In this study, modeling the optical scattering resulted in improved quantification.

Production of protein therapeutics by mammalian cells is the most widely used bioprocess because of its ability to properly produce and fold a recombinant protein, with 60–70 % of biopharmaceuticals using this bioprocess [79, 80]. Since 1986, an increased understanding of cell biology, gene transfer mechanisms, media composition, and process control has resulted in significant improvements to cell viability and titer [80]. Most cell culture bioprocesses use CHO cells, fed by glucose. Glucose is a critical process parameter, as it affects the cell’s metabolic profile, production of waste products, and post-translational non-enzymatic glycosylation of proteins [81]. CHO cells are typically fed in a batch, known as fed-batch, where glucose is delivered into the bioreactor as a large bolus at set time points, and the time points are based on a priori process knowledge and off-line analysis. However, this approach is not ideal for several reasons. It is labor- and resource-intensive, increases the risk for contamination, and does not adequately control glucose and lactate in a cell culture bioreactor. Noninvasive, real-time PAT measurements combined with PID or closed-loop feedback control can optimize feeding strategies, improving yield and titer.

Raman spectroscopy has an important role in cell culture bioprocesses, providing in situ measurements and enabling real-time process control. In situ Raman measurements were first reported in 2011 by Abu-Absi et al. [82]. Raman spectra were collected every 2 h over the course of ~14 d in a 500 L bioreactor growing CHO cells. The Raman spectrum enabled simultaneous measurement of glucose, glutamate, glutamine, lactate, ammonia, total cell density (TCD), and viable cell density (VCD). Raman data models based on PLS regression accurately predicted changes in nutrient and byproduct levels, and correlated with reference or calculated values. Raman estimates of TCD and VCD correlated with reference data, with slight deviations observed at very low cell densities at d 0–2. Later studies extend the capability of the technique by demonstrating cross-scale model transferability within development scale from 3 to 15 L [83], and from development scale (3, 200 L) to clinical manufacturing scale (2000 L) [84]. In these models, it was assumed that the bioprocess could be modeled using a linear model.

Two Raman-based feedback control studies underscore the capability of Raman to not only provide in situ chemical information, but also control protein quality. In a feasibility study by Craven et al., Raman spectroscopy was integrated with a nonlinear model predictive controller (NMPC) [85]. Raman spectra were collected every 6 min in a 15 L bioreactor growing CHO cells. A PLS data model was used to predict glucose, glutamine, lactate, and ammonia. PLS-predicted glucose concentrations were input into a NMPC, which communicated with an OPC-controlled pump to adjust the feed rate. Raman data correlated with off-line reference measurements. This approach allowed the bioreactor to maintain glucose concentration of 11 mM throughout the bioprocess. Off-line simulation studies were first used to optimize the controller configurations, then applied to two PAT scenarios. The first PAT scenario mimicked a case where off-line measurements are collected, and the feed rate is adjusted once per day. The second PAT scenario mimicked a case where in situ measurements are collected throughout the day and enabled closed-loop feedback control. Overall, the NMPC demonstrated good performance despite slight process–model mismatches, high measurement noise, unexpected occurrences, and long measurement intervals. In another study, Berry et al. demonstrated that a simplified Raman model for glucose feedback control in a fed-batch CHO bioprocess using two short cell-free and eight bench-scale production bioreactors successfully reduced non-enzymatic glycosylation of the target protein from 9 to 4 % [81].

Off-line Raman spectroscopy of CHO fed-batch bioprocess fresh and spent media enabled glycoprotein yield prediction [86]. The batch scale varied from 1 to 5000 L, representative of laboratory and manufacturing scales. Raman spectra were analyzed by PLS using the fingerprint region (400–1853 cm^–1^), which resulted in high relative error of prediction (REP, 7.9–13.1 %) and poor correlations (R^2^ < 0.4). Then, Competitive adaptive reweighted sampling (CoAdReS) and ant colony optimization (ACO) variable selection techniques were used to select a limited number of variables, or spectral regions, to improve model accuracy. The use of CoAdReS and ACO resulted in a more accurate PLS model, as evidenced by a reduction in REP to 2–4 % and an R^2^ > 0.9 for CoAdReS and R^2^ > 0.85 for ACO.

## Chemometric modeling of bioprocesses

Spectroscopic sensors are sometimes called “soft sensors” in the bioprocessing literature because spectroscopic data is modeled in software programs and the models provide information akin to hardware sensors [87, 88]. The use of data analysis models is important in order to extract the maximum amount of information from Raman spectra, and there is considerable research in this area. A 2012 review by Lourenço et al. provides an overview of univariate and multivariate models used in bioprocessing, which include PLS and PCA [37]. Fermentation or cell culture bioprocesses involve complex data with limited first principles knowledge on the process. How does a data model derive meaningful information from such a dataset? There are two reported approaches: explicit and implicit models.

Explicit models are sometimes called “first principles,” parametric or hard models. A commonly known explicit model is the Beer-Lambert law, which describes the relationship between a material’s optical absorbance and concentration [89]. Explicit models describe the system in terms of measured independent variables that produce dependent variables and essentially force the data to obey a mathematical model. Use of the “explicit model” term has only been found in two Raman/bioprocessing papers, and the definition of an explicit model in this context means that an internal reference was used to account for physical variations such as laser attenuation or optical scattering [90, 91]. Even if the “explicit model” term was not necessarily used, other studies have reported using an internal standard to improve quantification. An explicit model was used in the earliest examples of Raman analysis of bioprocesses, using the weak water peak ~1640 cm^–1^ as an internal standard [72, 73]. This approach has been recently extended to in situ Raman monitoring of *E. coli* culture [90], confocal Raman microscopy of media [91], and yeast fermentation [78]. A 2007 report of yeast fermentation used the 980 cm^–1^ sulfate band as an internal standard [77]. Even when an explicit model is used, there are often limitations to the known model, which may affect its robustness. In the 2014 study by Iversen et al., the authors noted that the relationship of yeast cell concentration with light extinction could not be adequately described using Beer-Lambert law, that non-linear, wavelength-dependent attenuation necessitated describing the relationship for each individual component using quadratic equations, and that the modeling was valid because the yeast cells were the only Lorenz-Mie scattering particles in the bioprocess [78]. An explicit model for CHO cell culture fed-batch was developed to account for bioprocess nonlinearities, with demonstrated success for in-process control of glucose concentrations [85]. Correcting for turbidity in aqueous media is of interest for Raman spectroscopy of water quality applications, and the developed approaches may be adapted for bioprocessing [92].

Implicit models are also known as non-parametric or soft models. Implicit models are based on correlation or covariance, assume no knowledge about the physical laws of the system, nor assume variable independence, and do not force the data to fit a known model. Measured training sets span a representative range of analyte concentrations. Implicit models account for physical or process variations without necessarily modeling that variation, and most reported models are implicit in that they do not assume a physical description of the system. Implicit models are especially useful when there is spectral overlap of components, as is the case in cell culture bioprocesses.

Model robustness and transferability are important considerations. Generic models are developed in one process, comprising a set of cell lines, media, and process conditions, and are sufficiently robust so that they can be applied to other processes or other Raman instruments. In this aspect, explicit models have an advantage since they are generally more robust than implicit models. Transferability of implicit models relies on the training set, and may not be applicable to other systems. However, if the training set of an implicit model adequately captures process variability as a result of different cell lines, media composition, or bioreactor volume, it may be considered a “generic” model; implicit models have been successfully transferred, as demonstrated since 1999. Shaw et al. demonstrated that a multivariate model resulted in the least amount of prediction error, and was the first to show that a model developed for one process could be successfully transferred as long as the same process was used [93]. Cross-scale transferability of implicit models, for the same bioprocess, was demonstrated within the development scale and from development to manufacturing [83, 84]. A recent study by Mehdizadeh et al. successfully demonstrated the use of a generic PCA/PLS model for bioreactors involving CHO cell lines [94]. Model predictions of glucose, lactate, and viable cell density were shown to be adequate for independent validations at the large pilot scale and in a cell line that was not included in model development. To our best knowledge, there are no reports of cross-scale transferability of explicit models in bioprocessing.

There are examples of explicit and implicit models in the bioprocessing literature, and both model types are suitable for in situ bioprocess monitoring and control. Although the early studies of Raman in bioprocesses used an explicit model, the modern trend is toward an implicit model. There are practical and scientific factors that contribute to this observed trend. As soft models became more sophisticated, they became more useful in their predictive capability, which facilitated their adoption in modeling bioprocesses. The complexity of the system, combined with logistical and time constraints, may preclude development of an explicit model despite the strong benefit of an explicit model to explicitly account for biochemical and process variations. Hybrid models, also called grey models, combine hard constraints with soft model flexibility and several have been proposed for bioprocesses [85, 95–97]. We anticipate more basic research and applications incorporating novel chemometric models into bioprocessing, enabling even more sophisticated analyses.

## Conclusions

Emerging technologies, a dynamic regulatory landscape, and new scientific challenges continue to expand the applicability and utility of Raman spectroscopy as a PAT in pharmaceutical manufacturing, and bioprocessing. Technological developments in transmission Raman enable nondestructive and rapid bulk tablet or capsule analysis. Transmission Raman is used primarily for off-line measurements of content uniformity. In-line Raman spectroscopy for API reaction monitoring or secondary pharmaceutical processes has been shown to enable real-time process corrections. Extension of Raman to continuous manufacturing environments has been demonstrated since the 1990s, and we expect to see more applications in this environment. Although not discussed in this review, there is much research in developing enhancement techniques for Raman spectroscopy. Surface-enhanced Raman spectroscopy (SERS) can be a powerful tool for in-process measurements, and feasibility has been shown for SERS of bacterial analysis and in bioprocessing [98–102]. Raman is a valuable PAT for fermentation or cell culture bioprocess monitoring and control. Simultaneous, in situ measurement of nutrients, metabolites, and cell concentration is an attractive feature of Raman. Based on our experience and a recent conference presentation, Raman is used in bioprocess monitoring and control from the laboratory scale to GMP production. We anticipate new reports describing the integration of Raman into a GMP environment.
